# Descending Influences on Vestibulospinal and Vestibulosympathetic Reflexes

**DOI:** 10.3389/fneur.2017.00112

**Published:** 2017-03-27

**Authors:** Andrew A. McCall, Derek M. Miller, Bill J. Yates

**Affiliations:** ^1^Department of Otolaryngology, University of Pittsburgh School of Medicine, Pittsburgh, PA, USA

**Keywords:** vestibular nuclei, reticular formation, posture, balance, vestibulo-autonomic interactions, sympathetic nervous system

## Abstract

This review considers the integration of vestibular and other signals by the central nervous system pathways that participate in balance control and blood pressure regulation, with an emphasis on how this integration may modify posture-related responses in accordance with behavioral context. Two pathways convey vestibular signals to limb motoneurons: the lateral vestibulospinal tract and reticulospinal projections. Both pathways receive direct inputs from the cerebral cortex and cerebellum, and also integrate vestibular, spinal, and other inputs. Decerebration in animals or strokes that interrupt corticobulbar projections in humans alter the gain of vestibulospinal reflexes and the responses of vestibular nucleus neurons to particular stimuli. This evidence shows that supratentorial regions modify the activity of the vestibular system, but the functional importance of descending influences on vestibulospinal reflexes acting on the limbs is currently unknown. It is often overlooked that the vestibulospinal and reticulospinal systems mainly terminate on spinal interneurons, and not directly on motoneurons, yet little is known about the transformation of vestibular signals that occurs in the spinal cord. Unexpected changes in body position that elicit vestibulospinal reflexes can also produce vestibulosympathetic responses that serve to maintain stable blood pressure. Vestibulosympathetic reflexes are mediated, at least in part, through a specialized group of reticulospinal neurons in the rostral ventrolateral medulla that project to sympathetic preganglionic neurons in the spinal cord. However, other pathways may also contribute to these responses, including those that dually participate in motor control and regulation of sympathetic nervous system activity. Vestibulosympathetic reflexes differ in conscious and decerebrate animals, indicating that supratentorial regions alter these responses. However, as with vestibular reflexes acting on the limbs, little is known about the physiological significance of descending control of vestibulosympathetic pathways.

## Introduction

Historically, vestibular-elicited reflexes were considered to be stereotyped responses to particular head movements (1–3). However, recent research has shown that vestibular nucleus neurons receive inputs from a variety of sources in addition to the inner ear, such that vestibular-elicited reflexes are shaped in accordance with ongoing movements and behaviors. For example, the work of Cullen et al. showed that some vestibular nucleus neurons compare the planned head movement with feedback sensory signals, and discharge in accordance with deviations from the intended movement (4–6). However, most studies that considered the modification of vestibular responses in a behavioral context focused on the control of eye movements and gaze (4, 7, 8), but not vestibular reflexes acting on the limbs and vestibulosympathetic responses that regulate blood pressure.

This review considers the integration of vestibular and other signals by the central nervous system pathways that participate in balance control and blood pressure regulation, with an emphasis on how this integration may modify posture-related responses in accordance with behavioral context. It starts with an overview of the pathways that relay vestibular signals to limb motoneurons and sympathetic preganglionic neurons, and then considers the integration of signals that occurs in these pathways. Next, evidence is presented that descending signals modify vestibulospinal reflexes acting on the limbs as well as vestibulosympathetic reflexes, presumably to shape the responses in accordance with physiological challenges that are present or anticipated, as well as behaviors and movements that are planned or being implemented. Finally, directions for future research are discussed. In total, the review describes how perspectives on vestibulospinal and vestibulosympathetic responses have evolved, such that these responses are now considered to be components of a hierarchy of systems that are activated to achieve stable movements without disturbances in blood pressure.

## Organization of Vestibulospinal and Vestibulosympathetic Pathways

### Vestibulospinal Pathways Acting on the Limbs

Two pathways originating in the vestibular nuclei provide inputs to spinal cord segments containing limb motoneurons. The medial vestibulospinal tract (MVST) originates in the rostral portion of the descending vestibular nucleus as well as the bordering areas of the medial and lateral vestibular nuclei (9–11). The main influences of this pathway are on upper-body musculature, particularly neck musculature, although a small fraction of MVST projections provide inputs to segments containing forelimb motoneurons (10–12). The lateral vestibulospinal tract (LVST) originates mainly from the lateral vestibular nucleus, with some contribution from the descending nucleus (9–11). This tract extends the entire length of the spinal cord and provides extensive inputs to spinal cord segments containing motoneurons that innervate forelimb and hindlimb muscles (13, 14). Since the LVST provides much more extensive inputs to the spinal cord segments containing limb motoneurons than does the MVST (10–12), it likely plays a predominant role in controlling postural responses of the limbs. Thus, the LVST will be a focus of this article.

The LVST mainly terminates in Rexed’s laminae VII and VIII in the forelimb and hindlimb segments of the spinal cord, which contain premotor interneurons, and not directly on motoneurons (13–16). Electrophysiological studies have confirmed that most connections of LVST axons with limb α-motoneurons are polysynaptic, although some weak monosynaptic inputs may occur to hindlimb motoneurons (17, 18). These observations suggest that signals conveyed through the LVST are processed and likely modified by spinal interneurons prior to reaching motoneurons. The LVST mainly has excitatory effects on extensor motoneurons, with some inhibitory effects on flexor motoneurons (17, 18). At least in cats, approximately half of LVST axons that terminate in lower cervical and upper thoracic spinal segments (which contain forelimb motoneurons) also have a branch to the lumbar spinal cord, raising the prospect that some LVST neurons simultaneously control muscle activity in both the forelimbs and hindlimbs (19).

Neurons whose axons project to the spinal cord in the pontine and medullary reticulospinal tracts (RST) also receive extensive vestibular inputs (20–23). These vestibular inputs are polysynaptic, indicating that vestibular nucleus neurons and other pathways convey labyrinthine signals to RST neurons, but that RST neurons do not receive direct inputs from vestibular nerve fibers (20, 21). RST neurons have both excitatory and inhibitory effects on flexor and extensor forelimb and hindlimb motoneurons (24–28), as opposed to LVST neurons that tend to excite extensor motoneurons and inhibit flexor motoneurons (17, 18). However, LVST and RST (24, 25, 29) neurons are similar in that their effects on the excitability of limb motoneurons are mainly polysynaptic, *via* spinal interneurons, and that the axons of both RST (30) and LVST (19) neurons are highly branched. Thus, the two major pathways that convey vestibular signals to limb motoneurons have some similarities, as well as some major differences.

### Vestibulosympathetic Pathways

The first key study demonstrating that the vestibular system contributes to cardiovascular regulation was published by Doba and Reis (31). As discussed in detail in a recent review (32), considerable evidence from experiments in both human and animal subjects has shown that the vestibular system participates in regulating sympathetic nervous system activity, to provide for adjustments in blood pressure during movement.

**Figure 1:**
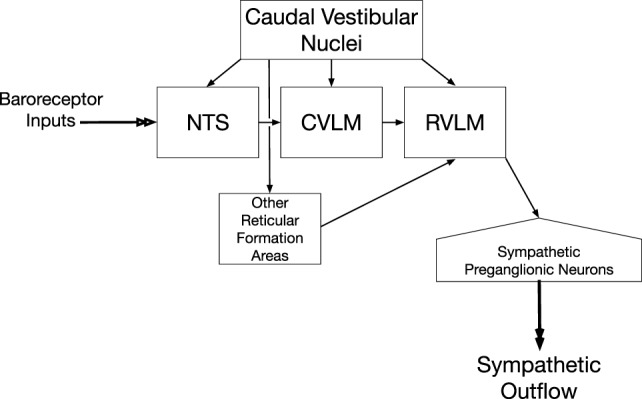
**Vestibulosympathetic reflex pathway**. Neurons in the caudal portions of the vestibular nuclei provide both direct and multisynaptic inputs to neurons in the rostral ventrolateral medulla (RVLM) with projections to sympathetic preganglionic neurons in the thoracic spinal cord. Multisynaptic pathways from the vestibular nuclei to the RVLM include relays in nucleus tractus solitarius (NTS), caudal ventrolateral medulla (CVLM), and other regions of the reticular formation including the medullary lateral tegmental field.

A column of reticulospinal neurons located near the ventral surface of the rostral medulla plays a predominant role in controlling sympathetic nerve activity and blood pressure (33–36). These neurons comprise the rostral ventrolateral medulla (RVLM). Lesions of the RVLM abolished vestibulosympathetic responses (37), suggesting that reticulospinal neurons with cell bodies in the RVLM constitute the major pathway through which vestibular signals are conveyed to sympathetic preganglionic neurons in the thoracic spinal cord. RVLM neurons are components of the baroreceptor reflex arc, which also includes neurons in nucleus tractus solitarius (NTS) that receive baroreceptor inputs, as well as inhibitory neurons in the caudal ventrolateral medulla (CVLM) (38–41). The RVLM receives direct inputs from the caudal aspects of the vestibular nucleus complex (42–47), as does the NTS (42, 48–51) and CVLM (42, 43, 45–47). The connections from the caudal portions of the vestibular nuclei to brainstem regions that regulate sympathetic nervous system activity are shown in Figure 1. In addition, neurons in other areas of the reticular formation that project to the RVLM, including those in the lateral tegmental field, receive labyrinthine inputs (52). Thus, RVLM neurons that control sympathetic nervous system activity receive both direct and multisynaptic inputs from vestibular nucleus neurons.

### Pathways for Dual Control of Sympathetic Outflow and Limb Muscle Activity

Although most studies have focused on pathways that independently regulate blood pressure and limb movements, transneuronal tracing studies demonstrated that neurons in the medial medullary reticular formation and adjacent raphe nuclei have collateralized projections to both sympathetic preganglionic neurons and limb motoneurons, as do some brainstem catecholaminergic neurons including those in locus coeruleus and nucleus subcoeruleus (53–56). As noted above, the activity of many medial medullary reticulospinal neurons is modulated by vestibular inputs (20–23). There is also evidence that medullary raphespinal neurons (57, 58), as well as spinally projecting neurons in locus coeruleus (59–61), receive vestibular signals. However, it is not yet known whether the dual-control neurons mediate both vestibulosympathetic and vestibulospinal responses. Moreover, the physiological role of the dual-control pathways is currently unknown, but these pathways should not be ignored when considering vestibular control of motor and sympathetic nervous system activity.

## Inputs to Vestibulospinal and Vestibulosympathetic Pathways

### Inputs to the Vestibular Nuclei

The vestibular nucleus complex on each side of the brainstem receives a wide variety of inputs. These inputs include vestibular signals from ipsilateral primary vestibular afferents and the contralateral vestibular nuclei, somatosensory inputs from the spinal cord, and modulatory signals from the cerebellum and higher-order brain centers. The convergence of widely dispersed inputs likely contributes, at least in part, to controlling the activity of vestibulospinal and vestibulosympathetic reflex pathways.

#### Vestibular Inputs

The central projections of vestibular afferents have been studied extensively and delineated in detail in prior reviews and chapters (8, 62), and thus will only be briefly described here. Vestibular afferents encode information about head tilt, translation, and rotations in space and project to the vestibular nuclear complex and to other areas of the nervous system that participate in balance control, such as the cerebellar nodulus and uvula (62–64). Vestibular afferents with peripheral processes innervating otolith and semicircular canal endorgans terminate ipsilaterally in all of the major vestibular nuclei, although projection patterns vary across species (62). Furthermore, the vestibular nuclei, excepting the lateral vestibular nucleus, are strongly interconnected by commissural projections from the contralateral side, and through ipsilateral intrinsic pathways (65, 66). Finally, inputs from multiple vestibular receptors, such as from otolith and semicircular canal inputs, converge onto single vestibular nucleus neurons, producing modulation of neural activity with varying spatial and temporal characteristics (termed spatiotemporal convergence) (67).

#### Spinal Inputs

Anatomic studies demonstrated that all levels of the spinal cord convey inputs to the vestibular nuclei (68–71). Cervical proprioceptive afferents send collaterals directly to the ipsilateral medial and inferior vestibular nuclei (68, 72, 73). Disynaptic pathways also carry afferent information from the cervical spinal cord to the medial, inferior, and lateral vestibular nuclei (74). Neurons within the lumbar enlargement send direct projections to the medial and inferior vestibular nuclei, and possibly the lateral vestibular nucleus (69, 70). Other indirect pathways, such as through the reticular formation and cerebellum, may also convey information from the spinal cord to the vestibular nuclei (70). Neurophysiologic studies conducted in a variety of species (mice, rat, cat, and cynomolgus monkey) and preparations confirmed that stimulation of somatosensory afferents from the neck and limbs modulates the activity of vestibular nucleus neurons (75–81).

#### Visual Inputs

Many neurons in the vestibular nuclei have activity that is related to eye position or eye movements [for reviews, see Ref. (4, 7, 8)]. Such neurons are not believed to contribute to vestibulospinal responses acting on the limbs and are largely absent from the caudal aspects of the vestibular nuclei that mediate vestibulosympathetic responses (82). Thus, these neurons will not be discussed further in this article. However, the activities of both neurons with eye-position sensitivity and “vestibular only” (VO) neurons that may contribute to vestibulospinal reflexes are modulated by movement of the visual field (optokinetic stimuli) (83–87). It is well-established that visual inputs contribute to postural control (88–95), and there is evidence that visual signals modulate vestibulosympathetic responses (96). However, it is unknown whether visual contributions to these responses are mediated through optokinetic inputs to the vestibular nuclei, or *via* other pathways.

#### Cerebellar Inputs

Cerebellar outflow to the vestibular nuclei is largely through the deep cerebellar nuclei, which are comprised of the fastigial, interposed, and dentate nuclei. Purkinje neurons in the cerebellar cortex send inhibitory projections to the deep cerebellar nuclei (97), which subsequently project to a variety of targets in the brainstem. The fastigial nucleus has been shown in a number of studies to project heavily to the vestibular nuclear complex (98–102); a few studies have also demonstrated projections to the vestibular nuclei from the interposed (103, 104) and dentate nuclei (105).

Some cerebellar cortical Purkinje neurons, particularly those in the vestibulocerebellum, project directly to the vestibular nuclei. Immunostaining studies localizing protein kinase C (PKC), an enzyme found in Purkinje cells, demonstrated that cerebellar Purkinje cells project to the major vestibular nuclei (106, 107). Anterograde and retrograde tracing studies showed that flocculus Purkinje neurons terminate in all of the major vestibular nuclei as well as group y, a cytoarchitecturally distinct extension of the vestibular nuclei located dorsocaudal to the restiform body (108–110). Tracing data also demonstrated that Purkinje cells in the nodulus send axons to the medial, inferior, and superior vestibular nuclei and to group y (111–113). Purkinje cells in the uvula were also shown to project to the superior and medial vestibular nuclei (112). The delta-isoform of PKC, which heavily stains Purkinje cells in the folia of the uvula and nodulus as well as the posterior cerebellum, is found in the caudal vestibular nuclei (inferior and medial vestibular nuclei), indicative of a significant direct cerebellar projection to these regions (107, 114).

#### Cerebral Inputs

Direct corticovestibular projections have been demonstrated in a number of animal preparations and are best described in cats and non-human primates (115–120), as reviewed by Fukushima (121). In the cat, injection of retrograde tracers into the vestibular nuclei labeled neurons is widely dispersed in areas of cerebral cortex including areas 6, 2, and 3a (115, 117). Electrical stimulation of areas 2 and 3a resulted in excitatory and inhibitory responses of vestibular nucleus neurons (115). This study also showed that vestibular nucleus neurons with spinal projections were particularly likely to have their activity modulated by cortical stimulation (115). Similarly, electrical stimulation of cortical motor areas (pericruciate cortex) resulted in short-latency (possibly monosynaptic) and long-latency excitation and inhibition of vestibular nucleus neurons (116). Studies in a variety of non-human primate species showed that widely dispersed cortical areas, including parieto-insular vestibular cortex, area 3aV, temporal area T3, premotor area 6a, area 7ant in squirrel monkey (corresponding to macaque area 2v), and the anterior cingulate cortex, project to the vestibular nuclei (119–122).

### Inputs to Reticulospinal Neurons

As previously discussed, reticulospinal neurons in the pons and medulla [pontomedullary reticulospinal tract neurons (pmRSTn)] that contribute to postural control receive extensive inputs from the vestibular nuclei (20–23). Although all four vestibular nuclei provide inputs to pmRSTn, the distribution, and extent of the projections from each nucleus are not uniform (21). Electrical stimulation of the VIII cranial nerve evoked disynaptic and polysynaptic excitation as well as polysynaptic inhibition of neurons in the pontomedullary reticular formation, confirming anatomical evidence that these cells receive extensive labyrinthine inputs (21, 123). A study in decerebrate cats indicated that pmRSTn receive extensive inputs from the otolith organs, which supports the notion that the RST plays an important role in generating gravity-dependent postural reflexes (22). However, some pmRSTn did not respond to stimulation of the VIII nerve, showing that the function of the RST is not simply to transmit vestibular signals to the spinal cord (21).

Extensive spinoreticular projections convey spinal afferent inputs to the reticular formation (124–128). As noted above, vestibular nucleus neurons receive extensive spinal inputs, such that projections from the vestibular nuclei to the reticular formation are another potential route for transmitting signals from skin and muscle to pmRSTn. Cutaneous inputs to the reticular formation are prevalent (124, 125, 128–131). Drew and Colleagues (131) reported that the majority of medullary RST neurons responded to cutaneous inputs, usually with an excitatory response. There is less evidence that pmRSTn receive inputs from muscle afferents, although one study in decerebrate cats showed that vibration applied to the gastrocnemius–soleus muscle complex altered the firing in 27% of neurons located within the nucleus gigantocellularis (a region of the medullary reticular formation) (132).

Like vestibular nucleus neurons, pmRSTn receive direct inputs from cerebral cortex (133–140). Inputs to pmRSTn are mainly from motor and premotor cortex, whereas vestibular nucleus neurons receive inputs from widespread cortical areas, at least in non-human primates (121). RST neurons, like vestibulospinal neurons, also receive extensive inputs from the cerebellar fastigial nucleus (141–143). However, unlike vestibulospinal neurons, pmRSTn receive little direct input from cerebellar Purkinje cells. Thus, there is a possibility that RST neurons are subject to different cortical and cerebellar control than vestibulospinal neurons, although this notion has not been thoroughly examined experimentally.

As noted above, some of the inputs to pmRSTn resemble those to vestibulospinal neurons. In addition, some reticulospinal neurons receive inputs distinct from those to the vestibular nuclei. For example, a population of spinally projecting neurons in the ventral and caudal aspect of the pontine reticular formation mediates acoustic startle responses (144). Such responses entail the stiffening of the dorsal neck, body wall, and limbs to provide protection from predatory attack before a defensive action can be engaged. The acoustic startle response entails monosynaptic inputs from the cochlear nuclei to reticulospinal neurons (145–148). Although vestibulospinal neurons may also mediate acoustic startle responses (149), the reticulospinal system appears to play a dominant role (150).

Shik et al. discovered in 1966 that electrical stimulation of a constrained region at the junction between the midbrain- and pons-elicited locomotion in animals (151). This area has subsequently been called the “mesencephalic locomotor region” (MLR) and has been identified across vertebrate species including man (152). The MLR does not project to the spinal cord, but provides inputs to pmRSTn (153, 154) that transmit locomotor signals to spinal premotor interneurons (155, 156). Hence, pmRSTn play a unique and pivotal role in controlling locomotion. There is no evidence that vestibulospinal neurons receive inputs from the MLR.

Although it is appreciated that a variety of inputs are conveyed to the reticular formation, little is known about the convergence of these inputs on single neurons (129). Moreover, only a few studies have entailed recordings from pmRSTn in conscious animals, or attempted to ascertain how the activity of these neurons changes during ongoing movements and across behavioral states.

**Figure 2:**
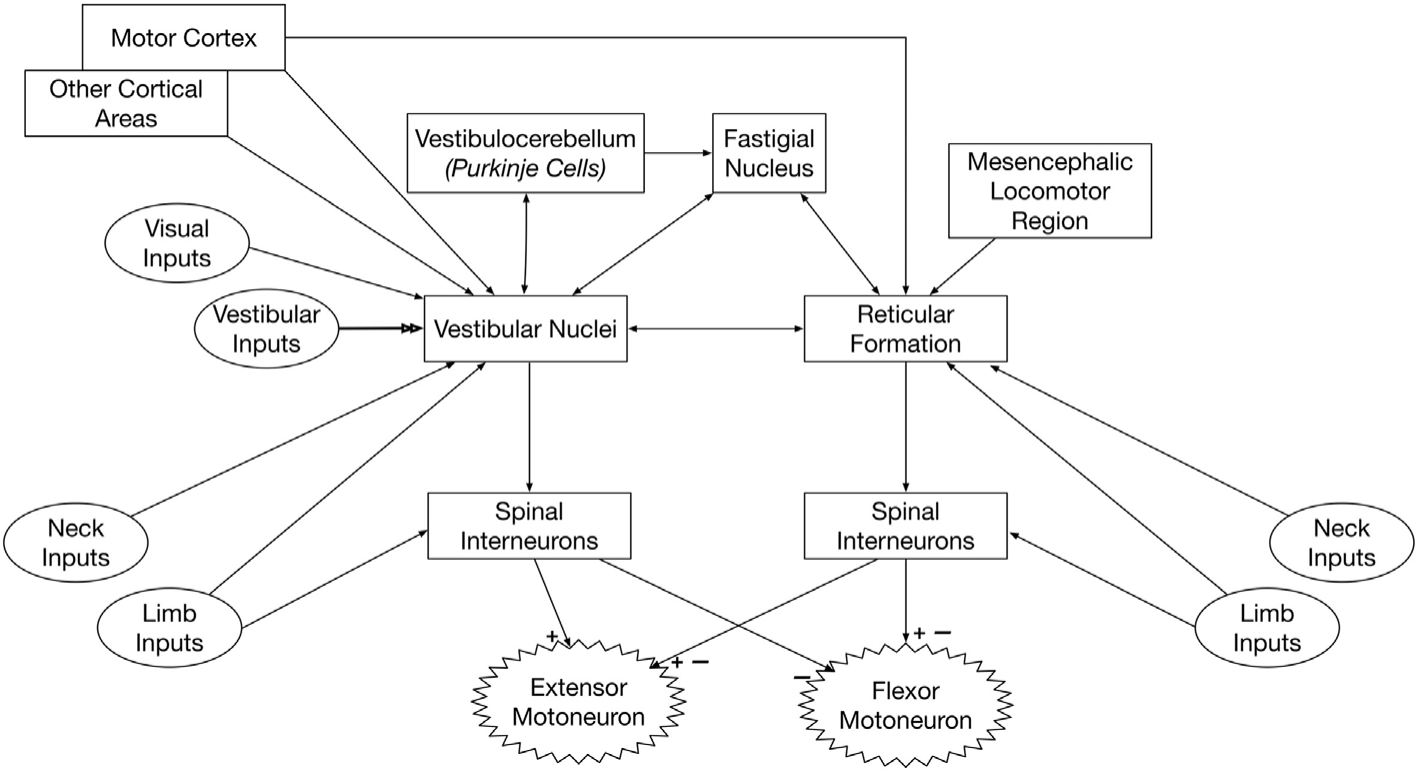
**Pathways that convey vestibular signals to limb motoneurons**. Spinal premotor interneurons receive inputs from the vestibulospinal and reticulospinal pathways. The vestibulospinal system excites extensor motoneurons and inhibits flexor motoneurons, while the reticulospinal system elicits both excitation and inhibition of flexor and extensor motoneurons. Thus, the spinal interneurons that receive inputs from vestibulospinal and reticulospinal pathways must be at least partially distinct. The neurons of origin of reticulospinal and vestibulospinal projections integrate vestibular, neck, and limb inputs; this integration is modulated by signals from several brain regions, including the cerebral cortex and cerebellum.

Figure 2 summarizes the inputs to and connections of vestibulospinal and reticulospinal neurons.

### Processing of Vestibulospinal and Reticulospinal Signals by Spinal Interneurons

As discussed above, few vestibulospinal and reticulospinal projections provide direct inputs to limb motoneurons, but instead mainly terminate on interneurons in Rexed’s laminae VII and VIII (24, 25, 29). A variety of identified types of spinal interneurons have been reported to receive labyrinthine inputs, including Renshaw cells (157, 158), Ia inhibitory interneurons (159, 160), propriospinal interneurons (161–163), and commissural interneurons (164–166). However, most experiments that characterized the responses of spinal interneurons to natural vestibular stimulation (whole-body rotations) did not identify the physiological role of those interneurons, or the convergent spinal and descending inputs they receive (167–169).

Considering the extensive projections of muscle spindle afferents to Rexed’s lamina VII (170, 171), it seems likely that there is convergence and integration of vestibular and proprioceptive signals in the spinal cord. A variety of descending pathways from the brain terminate in Rexed’s laminae VIII, including corticospinal, vestibulospinal, and reticulospinal projections [for review, see Ref. (172)]. A caveat is that the vestibulospinal system excites extensor motoneurons and inhibits flexor motoneurons (17, 18), while the reticulospinal system elicits both excitation and inhibition of flexor and extensor motoneurons (24–28). Thus, the spinal interneurons that receive inputs from the vestibulospinal and reticulospinal pathways must be at least partially distinct. Unfortunately, little is known about the convergence of signals from descending pathways in the spinal cord, and how the integration of the descending motor commands shapes ongoing and planned movements. Thus, the significance and physiological role of the processing of vestibular signals by spinal cord interneurons remain unclear. One role of the signal integration may be to adjust muscle activity when body orientation changes during locomotion, as while walking uphill (173–175).

It has also been suggested that the vestibulospinal and reticulospinal pathways provide inputs to gamma motoneurons, thereby affecting the gain of the myotatic reflex (176–178). However, some studies in animals (179), as well as recent experiments in humans (180, 181), indicated that vestibular signals do not affect the fusimotor system. Further experiments are needed to address the discrepancies in the conclusions of these studies.

### Inputs to Vestibulosympathetic Neurons

Neurons in the RVLM that control sympathetic nervous system activity receive vestibular inputs through a variety of pathways (32), as illustrated in Figure 1. Since many of the relays from the vestibular nuclei to the RVLM are polysynaptic, it is difficult to identify “vestibulosympathetic neurons” during neurophysiologic experiments. Thus, much of what is known about convergence of signals in the vestibulosympathetic pathways was ascertained by determining inputs and behavioral states that modulate sympathetic nerve activity elicited by vestibular stimulation.

Baroreceptors provide a dominant input to sympathetic nervous system neurons that control blood pressure, such that an increase in blood pressure results in a decrease in the activity of RVLM neurons as well as the sympathetic preganglionic neurons they provide inputs to (38–41). It is thus not surprising that stimulation of baroreceptors by increasing blood pressure resulted in an attenuation of vestibulosympathetic responses (182). A number of studies demonstrated the convergence of vestibular and baroreceptor inputs onto RVLM neurons, including putative presympathetic neurons with projections to the thoracic spinal cord (183, 184).

Rostral ventrolateral medulla neurons receive inputs from a variety of brain regions, either directly or indirectly through connections in the baroreceptor reflex pathway. These brain regions include the periaqueductal gray (185), parabrachial nucleus (186–193), several hypothalamic nuclei (190, 194–215), the amygdala (195, 212, 216–219), and prefrontal and insular cortices (206, 212, 220–225). Thus, engagement of a wide variety of descending pathways could potentially modulate vestibulosympathetic responses.

## Transformation of Vestibular Reflexes by Descending Pathways

### Transformation of Vestibulospinal and Reticulospinal Reflexes: Evidence from Animal Studies

The present body of knowledge regarding descending control of vestibulospinal reflexes largely stems from one of two experimental designs: comparing vestibular nucleus neuronal responses during active and passive movement in intact animals and comparing vestibular nucleus neural responses between decerebrate and intact preparations. While additional studies are clearly needed, in particular to discriminate which higher centers are responsible for modulating vestibulospinal reflexes, some insights can be gained from the present body of knowledge.

A subset of vestibular nucleus neurons identified in non-human primates, termed VO neurons, are thought to mediate vestibulospinal reflexes (226–229). The activity of VO neurons, like other vestibular nucleus neurons and primary vestibular afferents, is modulated by passive (externally applied) head movement with respect to space. However, unlike other classes of vestibular nucleus neurons, the firing rate of VO neurons does not change during eye movements (230, 231). During active (self-generated) head movement, responses of VO neurons are dramatically attenuated (227, 231, 232). Furthermore, proprioceptive feedback must match that signaled by the active motor command (efference copy) to suppress VO neuronal activity during self-motion (233, 234). While the locations of the circuits that compare efference copy with proprioceptive feedback during a movement have not yet been fully elucidated, descending inputs from higher brain centers must play a key role since volitional movement is triggered from cerebral cortex. In addition, it is unknown whether VO neurons responsible for reflex posturing of the limbs respond differently to active and passive (unexpected) limb movements.

Decerebration results in a disconnection of brainstem centers, including the vestibular nuclei and reticular formation, from higher brain centers. In decerebrate animals, the neural circuitry of vestibulospinal pathways is simplified by removing descending cortical influences, permitting investigations in a reduced preparation. The interruption of supratentorial inputs to the lateral vestibular nucleus is thought to produce unsuppressed activation of extensor motoneurons by the LVST, resulting in decerebrate extensor posturing (235–237).

As previously discussed, there is widespread convergence of afferent inputs from multiple sources, including vestibular, somatosensory, and visual signals, in the vestibular nuclei. The effects of hindlimb somatosensory inputs on the activity of vestibular nucleus neurons have been studied in both decerebrate and conscious cats by the same investigators, using the same equipment and methodology during experiments in both preparations, thus permitting comparisons (75, 78, 238, 239). Comparisons in decerebrate and conscious cats of the effects of electrical stimulation of hindlimb nerves on vestibular nucleus neuronal activity revealed some similarities, as well as differences (238, 239). Similarities across preparations include the following: the majority of vestibular nucleus neurons received convergent limb inputs from multiple nerves; response latencies were ~20 ms suggesting that polysynaptic pathways conveyed limb inputs to vestibular nucleus neurons; most responses were excitatory; and the proportion of neurons activated by hindlimb nerve stimulation increased after bilateral labyrinthectomy (238, 239). By contrast, low-intensity stimuli [<twice threshold (T) for eliciting a compound action potential in the stimulated nerve] elicited changes in activity of many vestibular nucleus neurons in decerebrate animals, but such low-intensity stimuli were ineffective in conscious animals. Only high-threshold stimuli (≥3 T) altered the activity of vestibular nucleus neurons in conscious animals. This finding suggests that supratentorial brain regions may suppress the transmission of inputs from large diameter hindlimb afferent fibers (i.e., group Ia and II afferents) to the vestibular nuclei in the conscious animal, or may block the responses of vestibular nucleus neurons to these signals.

**Figure 3:**
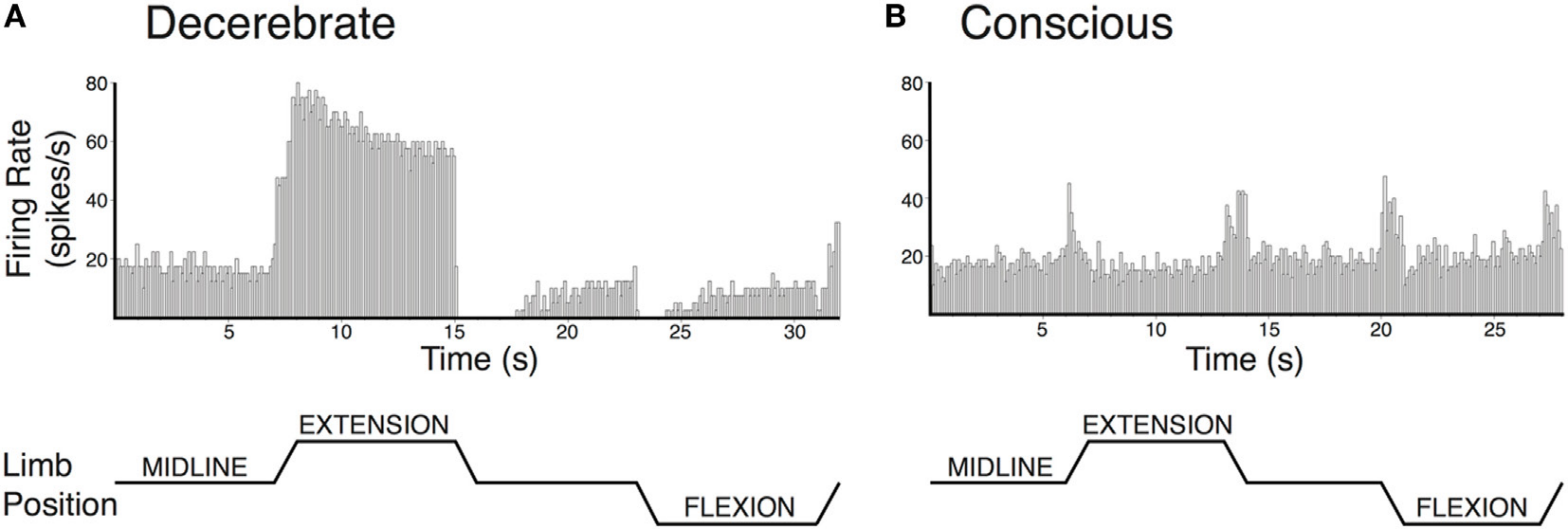
**Vestibular nucleus neurons responded to hindlimb movement in decerebrate (A) and conscious (B) cats, but the responses had different characteristics in the two preparations**. Vestibular nucleus neuronal responses to hindlimb movement in the decerebrate cat typically included encoding the direction of hindlimb movement and encoding hindlimb position signals. For example, the firing rate of the vestibular nucleus neuron depicted in panel **(A)** increased with hindlimb movements toward extension and decreased with hindlimb movements toward flexion. Furthermore, when the hindlimb was held in extension, neuronal firing remained elevated. While neurons with such response characteristics were also present in conscious cats, they were much less common than in decerebrate cats. By contrast, the activities of most vestibular nucleus neurons in conscious cats with responses to hindlimb movement were modulated in a similar fashion during all tested directions of hindlimb movement. For example, the activity of the vestibular nucleus neuron depicted in panel **(B)** increased with each hindlimb movement (midline to extension; extension to midline; midline to flexion; and flexion to midline). In both panels **(A,B)**, hindlimb movements were passively generated (externally applied) at 60°/s, lasted for a duration of 1 s, and were isolated to movements about the hip and knee joints. The hindlimb was held in each position for 7 s in panel **(A)** and 6 s in panel **(B)**. Unit activity was binned in 0.1 s intervals and averaged over four repetitions in panel **(A)** and eight repetitions in panel **(B)**. Panel **(A)** was adapted from Ref. (78); panel **(B)** was adapted from Ref. (75). Both panels were used with permission of the American Physiological Society.

Modulation of vestibular nucleus neuronal activity in response to hindlimb movement has also been compared in decerebrate and conscious cats (75, 78). While vestibular nucleus neurons responded to hindlimb movement in both preparations, some important differences were notable (see Figure 3). In decerebrate cats, the majority of vestibular nucleus neurons whose activity was modulated by hindlimb movement encoded the direction of hindlimb movement, and most also encoded hindlimb position signals (78). By contrast, most vestibular nucleus neurons in conscious cats encoded limb movement irrespective of the direction of the movement and did not encode hindlimb position (75). These findings suggest an interesting parallel with that noted above for experiments that made use of electrical nerve stimulation. If large-fiber afferents, such as those from muscle spindles, are responsible for the directional and position-related responses noted in decerebrate animals, the near complete lack of such responses in conscious animals suggests that higher brain centers selectively suppress this afferent feedback.

Similar comparisons of responses to limb movement in decerebrate and conscious cats have been performed for reticular formation neurons (240). In both preparations, the majority of responsive reticular formation neurons encoded limb movement irrespective of the direction of the movement; few encoded limb position or the direction of limb movement. In other words, the responses of reticular formation neurons to limb movement in both decerebrate and conscious animals resembled those of vestibular nucleus neurons in conscious animals. No suppression of limb position signals to reticular formation neurons by supratentorial brain regions was observed (240), as noted for vestibular nucleus neurons (75). These findings raise the possibility that higher brain areas such as cerebral cortex play fundamentally different roles in regulating the activity of the vestibulospinal and reticulospinal systems, although there is no experimental evidence to suggest the nature of the differences in regulation of the two systems.

### Transformation of Vestibulospinal and Reticulospinal Reflexes: Evidence from Studies in Human Subjects

**Figure 4:**
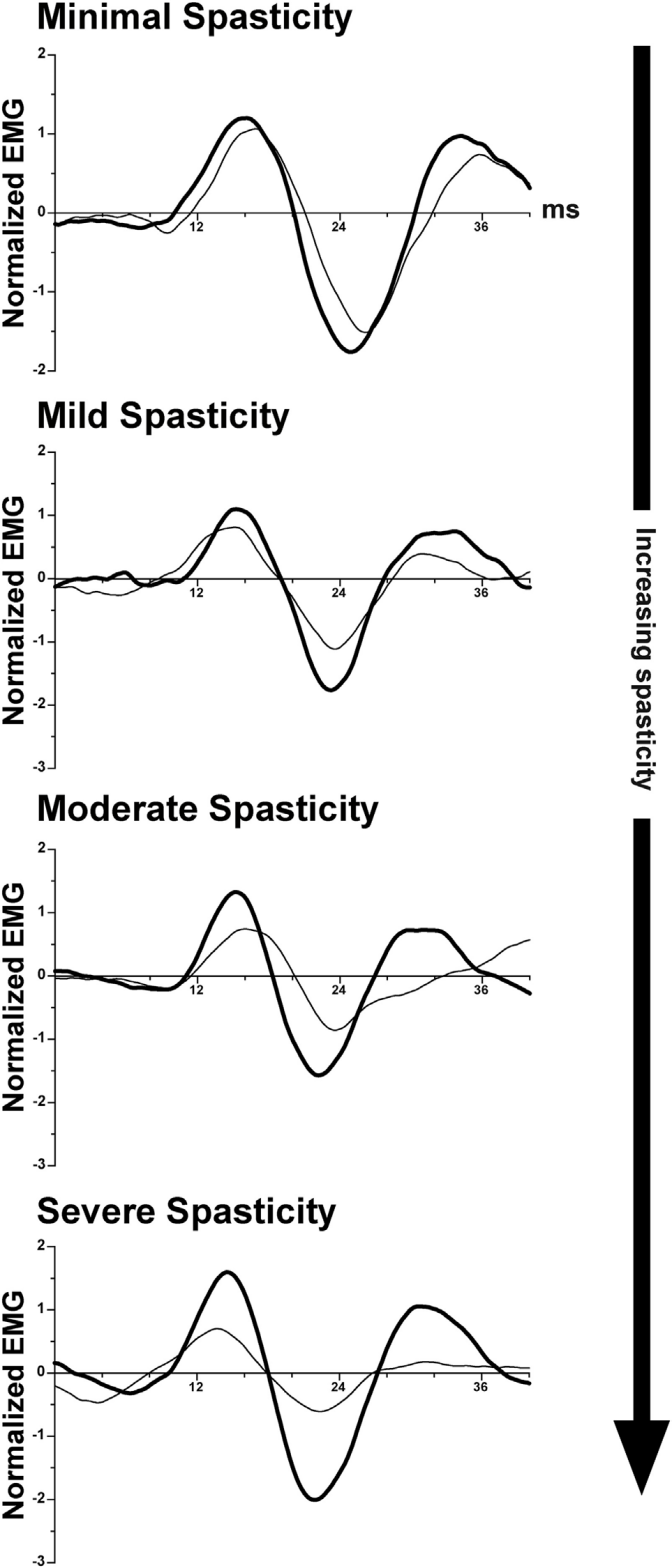
**Representative vestibular-evoked reflex responses from four subjects rank ordered as a function of increasing severity of spasticity**. Cervical vestibular-evoked myogenic potentials (VEMPs) were measured from the sternocleidomastoid muscles. In most subjects, the VEMP responses on the spastic-paretic side (thick line) were larger than on the contralateral side (thin line). As the degree of spasticity increased, so did the asymmetry in response amplitude between the two sides. Adapted from Ref. (248); used with permission of Elsevier.

As discussed above, transecting the midbrain in animals produces unsuppressed activation of extensor motoneurons, resulting in decerebrate extensor posturing (235–237). In humans, strokes affecting the internal capsule, which damage corticobulbar projections, produce an analogous condition: muscle spasticity (241, 242). Spasticity manifests as a sharply lateralized increase in muscle tone with enhanced tendon jerks (243). Several studies suggested that spasticity in patients, like decerebrate rigidity in animals, results from increased activity of vestibulospinal pathways (244–246). This is in contrast to one study in cats, which provided evidence that spasticity and decerebrate rigidity are differentially mediated through vestibulospinal and reticulospinal projections (247). A recent study in hemispheric stroke subjects supports the notion that spasticity results from disinhibition of vestibulospinal projections. Responses of neck muscles to vestibular stimulation (cervical vestibular-evoked myogenic potentials) were compared on the intact and lesioned sides in stroke survivors with spasticity. The differences on the two sides were proportional to the severity of the spasticity (248); the responses on the lesioned side were amplified, as illustrated in Figure 4. In combination with data from animals discussed previously, these data support the notion that supratentorial regions of the brain regulate the excitability of vestibulospinal neurons.

### Transformation of Vestibulosympathetic Reflexes

Evidence that supratentorial brain regions affect the gain of vestibulosympathetic reflexes comes from a comparison of these responses in conscious and decerebrate animals (184). Whereas about half of RVLM neurons responded to 10–15° tilts in vertical planes in decerebrate felines, the activity of <1% of RVLM neurons was modulated by similar rotations in conscious cats (184). Large rotations are required to generate vestibulosympathetic responses in conscious animals, presumably because small-amplitude tilts (<40%) do not produce peripheral blood pooling that necessitates an increase in sympathetic nervous system activity (249, 250). It has been suggested that higher brain areas adjust the responsiveness of neurons in the vestibulosympathetic reflex pathway to vestibular inputs, so that the gain of the vestibulosympathetic reflex is appropriate for the ensuing movement or postural change (32). Recordings from conscious animals also provided evidence that following a bilateral labyrinthectomy, the gain of the baroreceptor reflex is adjusted by descending signals from supratentorial brain regions (251). As noted above, a number of supratentorial regions provide inputs to neurons that comprise the vestibulosympathetic reflex pathway, and it is unclear which of these regions participates in adjusting the response gain, and where along the pathway (vestibular nuclei, NTS, CVLM, RVLM) the gain adjustments occur.

The notion that sympathetic nerve activity is regulated by higher brain centers during movement is not new. In both animals and humans, adjustments in sympathetic nerve activity and alterations in the set point of the baroreceptor reflex are initiated when exercise begins (252, 253). The changes in the baroreceptor set point are needed to allow blood pressure to increase during exercise. The term “central command,” which was coined by Goodwin et al., refers to the parallel changes in autonomic nervous system activity that accompanies muscle contraction (254). Perhaps the best evidence for feedforward cardiovascular responses during movement comes from a study in paralyzed human subjects, who exhibited increases in blood pressure and heart rate that were graded to the intensity of imagined activity (255). In decerebrate or anesthetized cats, stimulation of regions of the lateral and caudal hypothalamus, fields of Forel, MLR, and midbrain ventral tegmental area elicit parallel changes in motor activity and cardiovascular responses (253, 256, 257). However, little is known about signal processing in these regions that leads to changes in sympathetic nerve activity and the baroreceptor reflex set point.

## Conclusion and Directions for Future Research

Although there is a considerable body of data regarding vestibular system contributions to maintenance of stable eye position, much less is known about vestibular reflexes that ensure postural stability and constant blood pressure during movement. This is likely because vestibulospinal pathways that act on the limbs and vestibulosympathetic pathways that affect the cardiovascular system are much more complicated than the three and four neuron arcs that mediate vestibulo-ocular reflexes (3, 8). An often-overlooked aspect of vestibulospinal reflexes acting on the limbs is that they are mediated through premotor interneurons in the spinal cord (24, 25, 29), and not *via* direct connections of vestibulospinal and reticulospinal neurons with motoneurons. Virtually nothing is known about the convergence of descending motor signals with proprioceptive inputs in the spinal cord, and the role that spinal interneurons play in transforming the motor commands (11). Since there is a potential for spinal interneurons to integrate signals from a variety of descending motor pathways, the interneurons could potentially play an appreciable role in recovery of function if one of the pathways (or inputs to the pathways) is eliminated. Thus, a research focus is certainly warranted on the spinal interneurons that are components of vestibulospinal reflexes that act on the limbs.

Although it has been recognized for decades that both reticulospinal and vestibulospinal neurons convey vestibular signals to the spinal cord, and that these pathways receive somewhat different inputs, it is not yet clear if there is a distinct functional difference between the two pathways. As noted above, it is possible that signals conveyed through reticulospinal and vestibulospinal pathways converge on the same premotor spinal interneurons (11), although this is presently unknown. The reticulospinal pathway may be comprised of a number of parallel pathways with distinct functional roles such as mediating locomotion (153, 154), startle responses (144), and postural stability. Thus, it may be misleading to consider the reticulospinal pathway as a single functional pathway. Moreover, there is a paucity of data regarding the activity of vestibulospinal and reticulospinal neurons in awake, behaving animals, and the lack of this information complicates the determination of the physiologic roles of the pathways.

An experimental hurdle that complicates experiments in conscious animals on vestibulospinal and reticulospinal pathways is the difficulty in identifying neurons that are components of these pathways. Experiments entailing microstimulation in the spinal cord to ascertain the projections of vestibulospinal and reticulospinal neurons are tedious in paralyzed decerebrate or anesthetized animals (19), and even more complicated in conscious animals, since spinal stimulation can induce movement and extraneous inputs to the central nervous system. Consequently, there is a tendency to consider “VO neurons,” which are known to have a projection to the spinal cord, as a single class of neurons with uniform properties (227–230). Yet it is known from experiments in paralyzed animals that the branching pattern of individual vestibulospinal neurons can vary considerably (15, 16, 19). Vestibulospinal neurons that affect neck muscle activity may have dramatically different inputs and changes in activity across behavioral states than those that affect limb extensor activity. Thus, it is crucial to develop new methodology to ascertain the projection patterns of vestibulospinal neurons whose activity is monitored during neurophysiologic experiments, and to discontinue the practice of assuming that all vestibulospinal neurons must have the same properties. The same considerations should be applied to experiments on reticulospinal neurons.

Neurophysiologic experiments on neurons that constitute vestibulosympathetic pathways are in their infancy, and just two studies (184, 251) have monitored in conscious animals the activity of RVLM neurons that play a key role in regulating sympathetic nervous system activity. Little attention has been paid to the contributions of other pathways to vestibulosympathetic responses, including those originating in the medial reticular formation, raphe nuclei, and locus coeruleus that provide monosynaptic or polysynaptic inputs to both limb motoneurons and sympathetic preganglionic neurons (53–56). These pathways were identified in transneuronal tracing studies in rodents, and it is unclear whether they exist in other species, and what their role is in autonomic and motor control. In addition, it is critical that neurophysiologic studies on these neurons include an identification of their projection patterns, to ascertain which outputs they control.

In conclusion, it is now well-demonstrated that vestibulospinal and reticulospinal neurons that contribute to postural stability, and brainstem neurons that adjust blood pressure during postural alterations, receive converging inputs from a variety of sources, including cerebral cortex. Accordingly, the activity and responses to stimuli of the neurons can vary tremendously in conscious and “reduced” preparations, such as anesthetized and decerebrate animal models. In the ongoing efforts of the scientific community to foster the reproducibility of experiments and their translation to treatments for patients, the differences in responses between preparations must be fully considered.

## Author Contributions

All the authors co-wrote the manuscript and co-prepared the figures, and approved the final version of the manuscript.

## Conflict of Interest Statement

The authors have no conflicts of interest to declare related to this manuscript. No third-party payments were received related to the contents, and the authors have no financial interests that influenced the writing of the article.
